# Glycogen storage disease type III: modified Atkins diet improves myopathy

**DOI:** 10.1186/s13023-014-0196-3

**Published:** 2014-11-28

**Authors:** Sebene Mayorandan, Uta Meyer, Hans Hartmann, Anibh Martin Das

**Affiliations:** Clinic for Paediatric Kidney-, Liver and Metabolic Diseases, Carl-Neuberg-Str.1, D-30625 Hannover, Germany; Present address: Department of Paediatrics, University Hospital Münster, Albert-Schweitzer-Campus 1, D-48161 Münster, Germany

**Keywords:** Glycogen storage disease, Modified Atkins diet (MAD), Ketone bodies, Hyperinsulinism, Cardiomyopathy

## Abstract

**Background:**

Frequent feeds with carbohydrate-rich meals or continuous enteral feeding has been the therapy of choice in glycogen storage disease (Glycogenosis) type III. Recent guidelines on diagnosis and management recommend frequent feedings with high complex carbohydrates or cornstarch avoiding fasting in children, while in adults a low-carb-high-protein-diet is recommended. While this regimen can prevent hypoglycaemia in children it does not improve skeletal and heart muscle function, which are compromised in patients with glycogenosis IIIa. Administration of carbohydrates may elicit reactive hyperinsulinism, resulting in suppression of lipolysis, ketogenesis, gluconeogenesis, and activation of glycogen synthesis. Thus, heart and skeletal muscle are depleted of energy substrates. Modified Atkins diet leads to increased blood levels of ketone bodies and fatty acids. We hypothesize that this health care intervention improves the energetic balance of muscles.

**Methods:**

We treated 2 boys with glycogenosis IIIa aged 9 and 11 years with a modified Atkins diet (10 g carbohydrate per day, protein and fatty acids ad libitum) over a period of 32 and 26 months, respectively.

**Results:**

In both patients, creatine kinase levels in blood dropped in response to Atkins diet. When diet was withdrawn in one of the patients he complained of chest pain, reduced physical strength and creatine kinase levels rapidly increased. This was reversed when Atkins diet was reintroduced. One patient suffered from severe cardiomyopathy which significantly improved under diet.

Patients with glycogenosis IIIa benefit from an improved energetic state of heart and skeletal muscle by introduction of Atkins diet both on a biochemical and clinical level. Apart from transient hypoglycaemia no serious adverse effects were observed.

## Background

Glycogen storage disease type III (GSD III) is an inherited metabolic disease caused by deficiency of the glycogen debranching enzyme amylo-1,6-glucosidase and results in the accumulation of abnormal glycogen (‘limit dextrin’).

Two clinical types of GSD III are known:

GSD IIIa with liver and muscle involvement and GSD IIIb only with liver involvement [1,2].

In GSD IIIa, cardiomyopathy may lead to considerable morbidity [3–5].

Hypoglycaemia can be prevented by frequent feeding of carbohydrate–rich meals, continuous enteral feeding or administration of uncooked cornstarch [6–8]. This regimen does not improve cardiac and skeletal muscle function [9]. Cardiac dysfunction is treated symptomatically. Recent studies have advocated a protein-rich diet [10–12] which has also been supplemented with ketone bodies [9]. Even enzyme replacement therapy in GSD III has been suggested underpinning the unmet need for an effective treatment [13]. Krishnani et al. published guidelines on diagnosis and management of GSD III advocating the prevention of fasting, frequent feeds with high complex carbohydrates or administration of cornstarch in children whereas adults may be treated by a low-carb-high-protein-diet [14].

While a carbohydrate-rich diet prevents fasting hypoglycaemia in most cases it may lead to energy depletion of skeletal and heart muscle by the following mechanism: The administration of high-carbohydrate food induces reactive hyperinsulinism with subsequent suppression of lipolysis, ketogenesis, gluconeogenesis and activation of glycogen synthesis [15]. Thus, fatty acids, ketone bodies and glucose as fuel for skeletal muscle and heart are depleted.

Suppression of insulin secretion seems desirable in GSD IIIa, however simple hypocaloric catabolism will result in hypoglycaemia. The eucaloric ketogenic diet results in increased blood levels of ketone bodies as alternative substrates for the brain. At the same time, elevated levels of ketone bodies and fatty acids serve as energy substrates for heart [16] and skeletal muscle. Thus, the energetic balance of muscles is improved. Compliance with the classical ketogenic diet is often hampered by unpalatability and the necessity of calculating the ratio of fat to protein and carbohydrate intake which is supposed to be 4 (3):1. To improve compliance, we suggest using a modified Atkins diet (MAD) where daily carbohydrate intake is limited to 10 g while free access to protein is allowed and fat intake is encouraged.

We report treatment of 2 boys with GSD IIIa by MAD.

## Methods

### Patient 1

The boy of Sri Lankan extraction was 9 years old at the initiation of MAD. He was diagnosed with GSD III at the age of 7 months due to motor retardation, myopathy, and hepatomegaly.

Initially, he presented with elevated liver enzymes (ALT 711 U/l, AST 733 U/l and y-GT 52 U/l) and an increased creatine kinase activity (CK) in serum (514 U/l). The patient suffered repeated episodes of hypoglycaemia with minimal blood glucose levels of 0.1 mmol/l. GSD III was suspected and confirmed by the absence of amyloglucosidase activity in blood cells. Elevated glycogen concentration in red blood cells of 20 mg/dl (normal range 0–10 mg/dl) was in line with this diagnosis. Mutation analysis showed homozygosity for the mutation c.4256dupC. Echocardiography revealed slight hypertrophic cardiomyopathy.

The patient was initially treated with intravenous glucose infusion subsequently maltodextrin was given by continuous enteral feeding at night to prevent hypoglycaemia at a rate of 7–9 mg/kg per min. Stabilization of blood glucose was finally achieved by dietary treatment with uncooked cornstarch, which cannot be used before the age of 6–8 months due to immaturity of digestive enzymes. In the following years, feeding of carbohydrate and protein-rich meals as well as uncooked cornstarch over night was continued.

This classical diet stabilized plasma glucose levels, but echocardiographic follow-up showed progression of left ventricular and septal hypertrophy as well as left ventricular outflow tract obstruction (LVTO) under medication with verapamil. Interventricular septal wall thickness increased to 1.4 cm and LVOT-gradient rose to 20 mm Hg. ECG (electrocardiography) revealed signs for biventricular hypertophy and abnormal repolarisation with elevation of ST-segments (0.5 mV). The boy showed compromised physical capacity with initial difficulties in climbing stairs. Sport activities at school were very limited; he could hardly walk a distance of 100 metres without interruption. Constantly elevated levels of CK in serum (up to 5,300 U/l) over several years indicated significant muscle involvement.

As cardiomyopathy rapidly progressed we tried a protein-rich diet with a daily protein allowance of 3 g/kg (19% of total energy intake). However, cardiomyopathy continued to progress.

### Patient 2

The second patient was a boy of Iranian origin who was 11 years old at the start of MAD. In early childhood, recurrent episodes of hypoglycaemia occurred. The boy showed retardation in growth and motor development. At the age of 10 years, he was referred to our hospital with severe muscle involvement. No sport activities were possible and the boy had even difficulties climbing stairs due to muscle weakness. Moreover, he complained of chest pain after physical exercise and recurrent nausea. The glycogen concentration in red blood cells was elevated (23 mg/dl, normal range: 0–10 mg/dl), absence of amyloglucosidase activity in red blood cells confirmed the diagnosis of GSD III. The boy was homozygous for the mutation c.753_756del. Initial cardiac investigations revealed minor concentric hypertrophic cardiomyopathy with a prolonged QTc-time.

### Modified Atkins diet

The patients were hospitalized for the initiation of the modified Atkins diet for 7–14 days. This allowed close monitoring of both patients and comprehensive dietary training of the patients and their families. MAD consisted of meals low in carbohydrate with a total of 10 g carbohydrates per day. Families were given exchange tables for rough estimation of nutrient intake and special low-carbohydrate food was suggested. Patients were encouraged to eat food rich in fat and protein like meat, fish, eggs, nuts ad libitum. Vitamins and minerals were supplemented according to dietary recommendations. Compared to the classical ketogenic diet MAD is easier to implement in daily life as fat and protein intake do not have to be calculated which facilitates compliance. In ketogenic diet a 4:1 or 3:1 ratio of fat versus protein and carbohydrate has to be followed which involves calculating fat, protein and carbohydrate intake. While commercially available ketogenic formula feeds can facilitate daily life for infants during the first few months of life compliance is more difficult in the older child.

Occasional asymptomatic hypoglycaemia occurred in the first weeks after introduction of MAD thus uncooked cornstarch had to be continued. In patient 1 uncooked cornstarch at night could be slowly reduced over a period of 5 months and was then discontinued, patient 2 received uncooked cornstarch for two months only.

Both patients were followed in our outpatient clinics (patient 1: 32 months, patient 2: 26 months after start of MAD) and were seen every 3–6 months.

To assess the metabolic status and therapy compliance the following parameters were recorded every 3 to 6 months: ketone bodies in plasma and urine, CK-levels in serum, liver function, acylcarnitine profile, amino acids in plasma, lipids and glucose in serum. Cardiac investigations via echocardiography and electrocardiography were performed every 3–6 months. At home, parents monitored ketone bodies in urine via dip stick testing.

While the family of patient 1 had no problems sticking to MAD, the family of patient 2 decided to stop MAD after 3 months and switched to traditional cooking. Patient 2 and his family were noncompliant due to language difficulties and lack of comprehension of the disease. There were many (psycho-) social problems as the family had refugee status. In the following months we were able to convince the family to resume MAD.

## Results

Dietary treatment with MAD was well tolerated apart from transient hypoglycaemia. In patient 1 carbohydrate intake dropped from 10 g/kg per day to 0.4 g/kg per day under MAD, protein intake increased from 3 g/kg per day to 7 g/kg per day while fat intake increased from 1.6 g/kg per day to 8 g/kg per day. In patient 2 carbohydrate intake under MAD was 0.5 g/kg per day, fat intake 6 g/kg per day and protein intake 5 g/kg per day. No dietary assessment was done in this patient prior to MAD introduction. In both patients dietary recommendations according to DACH (German-Austrian-Swiss association for nutrition) were met as judged by dietary protocols during MAD.

### Patient 1

Essential laboratory and echocardiographic parameters are summarized in Table 1. Plasma concentrations of ketone bodies ranged from 1.7 to 7.8 mmol/l. The CK activity in blood fell significantly under MAD. Cardiac function improved as judged by echocardiography and ECG. Left ventricular outflow tract (LVOT) obstruction significantly improved; the gradient decreased from 20 mm Hg before initiation of MAD to 5 mm Hg after 32 months of MAD, ventricular septum thickness was reduced from 1.4 cm to 0.8 cm in the same period while posterior wall thickness remained constant (Table 1). This improvement is reflected in NT-Pro BNP (N-terminal fragment pro brain natriuretic peptide)-levels which were very high and normalized under MAD. Before MAD, the ECG revealed biventricular hypertrophy and abnormalities in repolarisation with a ST-elevation of 0.5 mV. After 32 months of MAD, repolarisation normalized, ST-elevation disappeared, while signs for left ventricular hypertrophy were still observed.

**Table 1:** **Laboratory parameters and echocardiographic studies in patient 1**

	**Time**								
**Parameter**	**−6 m**	**−3 m**	**0 m**	**+4 m**	**+8 m**	**+12 m**	**+18 m**	**+24 m**	**+32 m**
**Metabolic monitoring**									
KB [mM]	-	-	-	5.6	7.8	5.9	4.5	3.0	2.7
CK [U/l] (<170)	2646	3400	2650	1735	670	602	621	668	599
**Lipids**									
LDL-C [mg/dl] (<130)	126	120	111	120	115	125	140	118	130
TG [mg/dl] (<140)	180	184	180	183	192	180	224	181	250
**Cardiac monitoring**									
ProBNP [ng/l] (<84)	555	-	603	-	-	-	-	-	72
LVOT-gradient [mm Hg]	20	18	20	20	13	8	9	7	5
PWT [cm]	0.8	0.8	0.8	0.8	0.7	0.7	0.7	0.8	0.7
VST [cm]	1.4	1.3	1.4	1.4	1.1	1.2	1.0	1.0	0.8

Start of MAD at 0 months (0 m).

Laboratory parameters in blood: KB: Ketone Bodies in plasma; LDL-C: LDL-cholesterol in serum; TG: Triglycerides in serum; ProBNP: N-terminal fragment pro brain natriuretic peptide in serum;

Echocardiography: LVOT-gradient: Left Ventricular Outflow Tract-gradient; PWT: Posterior Wall Thickness; VST: Ventricular Septum Thickness.

The patient showed an increase of stamina and is able to walk 500 metres without interruption. Weight gain and growth are commensurate with age.

Apart from transient asymptomatic hypoglycaemia no side effects were observed. LDL-cholesterol levels were in the reference range, triglycerides were slightly increased (Table 1).

### Patient 2

MAD resulted in elevated ketone bodies in plasma with a maximum of 7.8 μmol/l. A decrease of the CK-level from 3,895 to 2,846 U/l was detected over a period of two months. Chest pain after physical exercise and nausea disappeared. The boy gained more stamina. Subsequently, under dietary noncompliance and complete withdrawal of MAD, ketosis was lost. CK-levels increased to 5,192 U/l. Chest pain after physical exercise reappeared and a reduction of physical capacity was observed. No cardiac follow-up could be performed due to incompliance.

**Figure 1:**
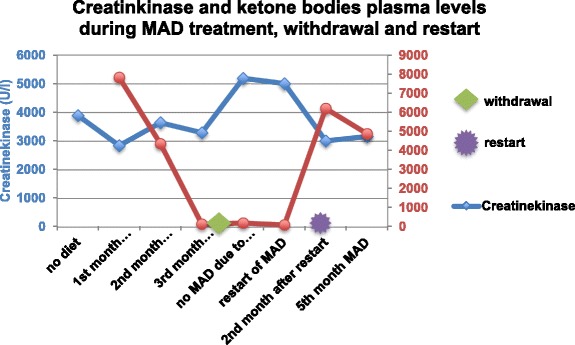
**CK- and ketone body levels in patient 2.**

When MAD was resumed ketosis was quickly re-established, CK-levels fell from 5,192 U/l to 3,000 U/l within 4 weeks (Figure 1). Chest pain and muscular weakness disappeared.

Hypertrophic cardiomyopathy disappeared, ECG normalized.

No serious side-effects resulting from MAD were observed, LDL-cholesterol and triglyceride levels remained normal (results not shown). Weight gain and growth were appropriate for age.

## Discussion

GSD III has classically been treated with frequent carbohydrate-rich meals. While this therapeutic option prevents hypoglycaemia it does not improve muscle [17] and cardiac dysfunction [18,19] including arrythmias [20].

We hypothesized that reactive hyperinsulinism resulting from high carbohydrate feeding is responsible for the depletion of energy substrates (fatty acids and ketone bodies) in skeletal and cardiac muscles followed by muscle dysfunction. Furthermore, elevated levels of insulin activate glycogen synthase and promote glycogen storage in myocytes [15]. This may exacerbate subtle energy deficiency which has been discussed to play a pathophysiological role in glycogenoses [21,22].

Our therapeutic approach using MAD led to clinical improvement/stabilisation of skeletal muscle and cardiac manifestations in both our patients. CK-levels in plasma as an objective quantitative parameter for muscle dysfunction dropped under MAD. Quantitative tests of muscle strength were not performed. A (involuntary) cross-over study took place in patient 2.

Heart function improved as judged by echocardiography and ECG in patient 1. Pro BNP as a marker for compromised heart function impressively improved under MAD. Detailed studies of heart function were not performed in patient 2 as heart function was not severely compromised before MAD was initiated.

Most likely, the underlying pathophysiological mechanism is prevention of hyperinsulinism and its consequences. Daily carbohydrate allowance was low in both patients at 0.4-0.5 g/kg which is sufficient to suppress insulin secretion. Adequate supply of energy substrates under MAD has a positive effect on muscle function. Furthermore, ketosis is known to activate mitochondrial succinate dehydrogenase in heart thus improving the energetic balance [23]. Reduction of the glycogen synthase activity with consecutive reduction of stored glycogen may be another beneficial factor. We observed transient hypoglycaemia at the start of MAD when ketone bodies were not yet elevated. Potential side effects of MAD as gastrointestinal symptoms, fatigue and dyslipidaemia [24] were not observed in our patients. So far, it is not clear what is necessary in terms of plasma ketone body concentrations to secure adequate energy supply for heart and skeletal muscles.

It is not clear why the response to MAD in patient 2 was less pronounced as judged by CK-levels. Response may depend on the specific mutation underlying GSD IIIa, alternatively dietary compliance may have been incomplete between visits in our outpatient clinics.

A similar stabilization and reversal of GSD IIIa-related cardiomyopathy and myopathy of respiratory muscles has been reported in patients on a high-protein diet [10–12] which is also suggested in a recent guideline [14]. We speculate that the underlying mechanism of this observation may be the prevention of reactive hyperinsulinism as well. Valayannopoulos et al. reported a case with improvement of cardiomyopathy under high-protein diet combined with administration of D, L-3-Hydroxybutyrate. The supply of exogeneous ketone bodies is unphysiological and requires compliance of the patient. Sticking to a ketogenic diet is more demanding than following MAD. Thus, MAD may be more efficient and comfortable for the daily routine of the patient.

## Conclusion

In summary, we report 2 boys with GSD IIIa who benefited from MAD. CK-levels fell, cardiac function improved and exercise tolerance increased. Apart from transient asymptomatic hypoglycaemia at the initiation of MAD no serious adverse effects were observed.

### Consent

Written informed consent was obtained from the parents of patient 1 and the mother of patient 2 for the publication of this report.

## Abbreviation

CK: Creatine kinase
CK-MB: Creatine kinase - MB
GSD III: Glycogen storage disease type III
LVOT: Left ventricular outflow tract
MAD: Modified Atkins diet
Pro BNP: N-terminal fragment pro brain natriuretic peptide
